# Making Older Adults' Cognitive Health Visible After Covid-19 Outbreak

**DOI:** 10.3389/fpsyg.2021.648208

**Published:** 2021-06-14

**Authors:** Francesco Della Gatta, Chiara Terribili, Elisa Fabrizi, Carmen Moret-Tatay

**Affiliations:** ^1^Escuela de Doctorado, Universidad Católica de Valencia San Vicente Mártir, València, Spain; ^2^Department of Neuroscience, Mental Health and Sense Organs (NESMOS), Faculty of Medicine and Psychology, Sapienza University of Rome, Rome, Italy; ^3^MEB Lab (Mind, Emotion and Behavioural Laboratory), Faculty of Psychology, Universidad Católica de Valencia San Vicente Mártir, València, Spain

**Keywords:** older adults, gender, cognitive health, COVID-19, lockdown, metacognition

The Covid-19 pandemic has brought unprecedented challenges to humankind. Community-dwelling older adults are considered one of the most vulnerable profiles and their chronic conditions might be aggravated by the consequences of lockdown (Burrai et al., 2020; Cordellieri et al., 2021; Pongan et al., 2021). Innovative approaches, supported by evidence disaggregated by age but also gender and relevant socio-economic characteristics, are crucial to effective public policy making that is inclusive for them. According to the WHO Department of Gender (2002), gender is used to describe the characteristics of women and men that are socially constructed. While gender perspective in the measures taken to combat Covid-19 outbreak should ensure gender equity for community-dwelling older adults, training certain individual factors might be an opportunity to address, or at least slow down, the negative effects of the current health crisis. In this way, several recent findings suggest that targeting metacognition skills might play an important role not only on technology adoption, but also in anxiety (Tsumura and Robertson, 2017; Capobianco et al., 2020). From an exploratory perspective, we discuss the effects on other cognitive processes, such as visuospatial navigation, one of the skills that might have suffered most from the effects of the restriction measures during covid-19.

The training of metacognitive awareness as a strategy might be of interest to address not only deficits underlying Cognitive Health, but also the gender gap described in previous literature, especially for older adults.

## Risks to Older People in COVID-19 Response

Among the prevention measures, social distancing seems to be the best way to slow down the silent spread of the Covid-19 virus. However, these measures, that not only have differences across countries, also keep most of its side effects that may arise in short, or even long term, unknown (Carbone et al., 2020; Murphy and Moret-Tatay, 2021). To date, most countries continue to discuss the implementation of protection rules for high-risk groups, such as the older adults, even after the end of the current regulation sand the increase in the number of vaccinations.

Not surprisingly, lockdown has been linked to physical and mental health, increasing the prevalence of symptoms of anxiety, depression, and feelings of loneliness during the first wave (Van Bavel et al., 2020; Pérez-Mengual et al., 2021). Literature in age-related differences has depicted paradoxical results over these lines. Even if older adults might be experiencing higher fear because of the vulnerability, some research indicate that they do not feel more anxiety or loneliness due to isolation and health conditions (Luchetti et al., 2020). This point is of interest for the study of ageism. Nevertheless, one should not forget that social isolation is also associated with increased risks of cognitive impairment, increasing the risk of developing neurocognitive disorders or accelerates the progression of pre-existing diseases (Baschi et al., 2020).

Gender plays a relevant role in this scenario. Older women role often provide care for older relatives and raise and care for children, which might result in an anxiety increase, as depicts literature (Ausín et al., 2020). How to recognize deficits and the search for solutions is an exercise of metacognitive awareness that can fortunately be trained in this population particularly to address gender divide (Hertzog and Dunlosky, 2011). Moreover, regarding communities or single-family group, many seniors depend on other people for their daily activities, while others are fundamental pillars for their families. Consequently, distancing required from the first wave might make those who do not use technology feel more isolated, and a gender gaps have been also described on technology adoption (Mitzner et al., 2019).

Social disconnection of the older adults also must be taken into consideration as an imperative variable, as this is the least adapted group to digital technologies (Moret-Tatay and Murphy, 2019; Moret-Tatay et al., 2019). Of note, older adults are commonly described as late users of technology compared to younger ones. They have been labeled as *digital immigrants* as they had to immigrate from analogy to a digital world in a relatively short time and come across mobility restrictions to stop the spread of a pandemic. Even if technology adoption is a variable of interest for older adults, this could be a double-edged sword type of risk. At the same time, it is important not to allow to exacerbate the emergency-feeling without considering the quality of the information given, as the increasing amount of fake news through social networks has its devastating effects within the Covid-19 pandemic. This false information could lead to serious harm such as raising anxiety levels, especially for older adults during lockdown (Dobson-Lohman and Potcovaru, 2020; Vahia and Shah, 2020).

In this context, the term digital gender divide is frequently used to refer to these types of gender differences in Information and communication Technology (ICT) adoption, which are fueled by digital illiteracy and reflected by a lack of comfort in using technology. These results are of interest for policy making that aim to ensure gender equity. The ability in which women access and use digital technologies is directly and indirectly affected by market related factors including investment dynamics, as well as regulations. This justification is supported by results in the literature that point out that gender gap in digital technology adoption in rural areas is more pronounced than in industrialized economies, as described by the World Wide Web Foundation in 2016. Not intervening in this type of gap would be like denying digital language and its benefits to this group. Differences related to socioeconomic status and gender are also a variable of interest, as the digital divide is higher among socially disadvantaged groups. In addition, education levels seem to generalize a gap in these levels (Joshi et al., 2020). Thus, to understand how to re-connect with others and keep them active seems imperative, from the strict lockdown to more relaxed distancing measures.

Literature has showed the obvious implications of restriction measures on mental health (Rossi et al., 2020). Its effects might be classified into three different levels: individual, familiar or community, and society. These are not isolated dimensions, as they can be overlapped. In this way, would it be possible to mitigate the detrimental effects of the current situation by working on individual variables?

## The Role of Metacognitive Awareness

**Figure 1:**
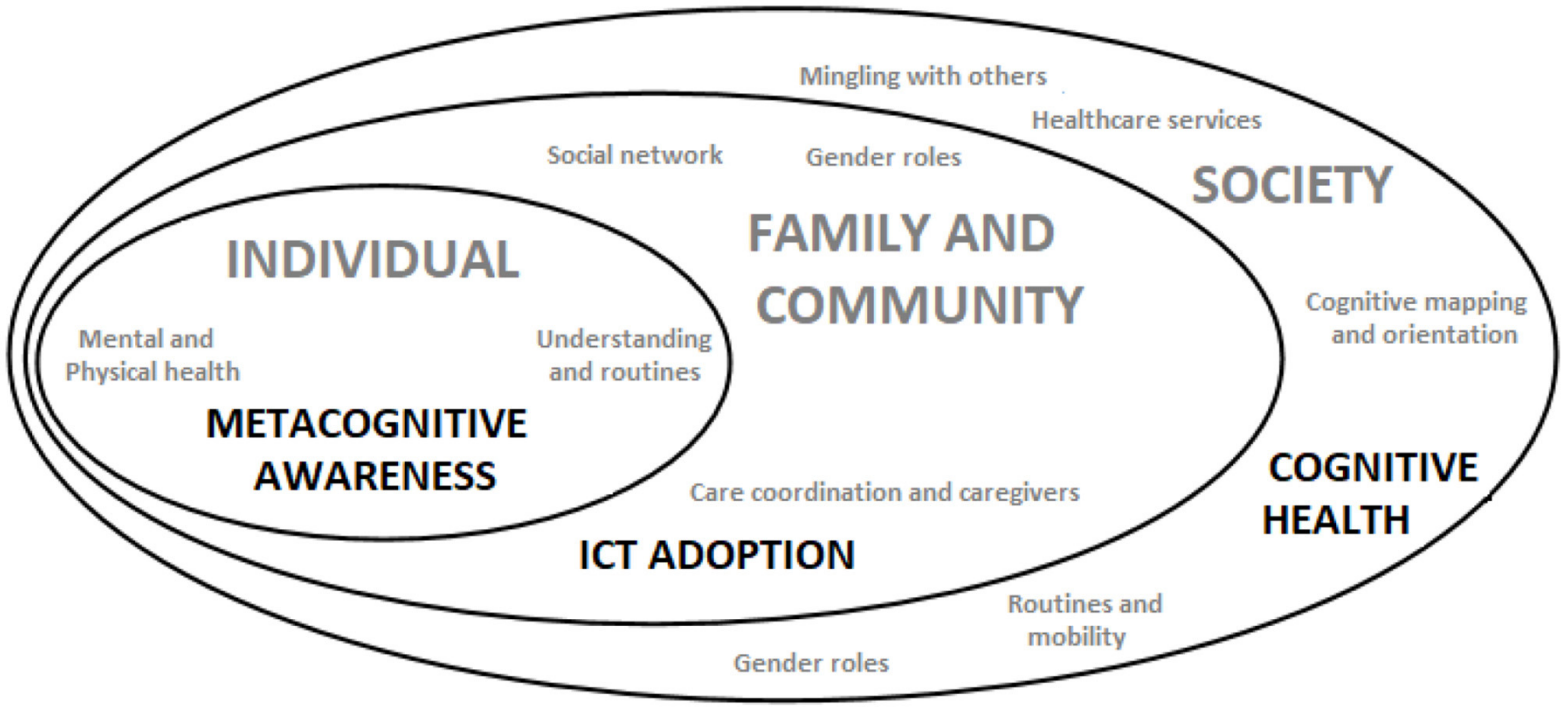
A schematic approach across the individual, the family and community, and the system and society variables in older's cognitive health, to keep the individual independent.

From a cognitive perspective, society requires an adequate approach to challenges such as social interactions, accessing and using of primary services for older adults. The role of cognitive health might emerge over these lines. According to the NIH National Institute on Aging (NIA) (2020), Cognitive Health is the ability to clearly develop basic cognitive processes and it is an important component in the aging process, as depicted in Figure 1.

In this context, metacognition is defined as an individual ability to understand one's cognitive skills, involving cognitive processes such as memory, attention, and problem solving (Duckworth et al., 2009). Some of them are considered components of executive functioning, which might be affected by mobility restriction in both physical and mental levels.

Metacognition is more than a term commonly defined as *thinking about thinking*. Likewise, metacognitive development would help to regulate cognition and to succeed both in lifelong learning and in conquering the ordinary rigors of everyday life (Zhao and Ye, 2020), especially in times of crisis such as the current pandemic. The training of metacognitive strategies is strongly linked to Cognitive Health in the aging process. This training might slow down the deterioration of certain crucial processes for seniors as indicated by other longitudinal programs (Farina et al., 2020a,b). Particularly, it is of interest for the adoption of digital technology, as previously stated, but also for other instrumental processes, such as the visual-spatial navigation.

Of note, while keeping routines is directly related to executive functioning in different levels, being able to navigate is linked to cognitive strategies for Cognitive Health in a society level, and wherever they may be a new or a familiar environment, it is a fundamental for daily routines. Individuals who struggle in this process will require external support to do so (such as caregivers or technological assistance). Likewise, human spatial navigation can be achieved through two major cognitive strategies: an egocentric (imagining location in reference to the self) and allocentric one (imagining location in reference to other objects or locations). Both strategies co-exist, and people, who have intact navigation skills, may interchange from one strategy to another depending on the best approach for the given situation. Map learning might influence allocentric knowledge, while route learning the egocentric one, supporting the idea that they might be partially dissociable in humans, even if they are learned in parallel (Boccia et al., 2014). The allocentric strategies would be affected by periods of confinement, favoring the predominance of egocentric strategies, which in turn would be related to a symptom of cognitive impairment. Throughout the aging process structural changes occur within specific areas of the brain that can negatively affect human navigation. These include a decrease in the frontal structures of the brain and the entire striatum (typically affecting egocentric strategies) as well as a reduction in the volume of the hippocampus (associated with allocentric strategies and generally spatial learning).

Metacognitive strategies training might support the use of allocentric strategies, particularly across gender. Once again, literature found a male advantage for navigation in virtual environments (Coutrot et al., 2019), and a gender advantage has been also described for men (Verde et al., 2015). In order to make visible questions of vulnerability, further research investigating how lockdown effects might affect gender roles on cognitive strategies for human spatial navigation is needed.

## Final Thoughts

The discovery of the detrimental cognitive impacts on the human brain of older adults after the global lockdown, are visible promptly in an interdisciplinary way. Of note, all these changes occur in a context where many areas have struggled to address the interaction of technology in a rapidly aging society.

A potential tool and strategy would be the awareness of metacognitive skills and training cognitive strategies from an individual perspective, while promoting digital technology adoption (reducing anxiety during this process) and support to families and community. Although, metacognition has been carefully investigated in the context of verbal learning, the literature in the non-verbal field is less common. Current research in neuroimaging and biomarker studies is focused on the search for cognitive fingerprints for the identification of cognitive impairment (Verghese et al., 2017), highlighting the role of navigational and spatial orientation deficits, or in other words, cognitive mapping. These are increasingly shown to be present in individuals at risk and it is one of the first signs of Cognitive impairment. It should be noted that spatial navigation might be one of the cognitive processes that suffers more during lockdown. Moreover, it has fewer verbal, cultural and educational biases than current cognitive tests and may allow a more uniform and comprehensive approach to cognitive fingerprinting. In this regard, work on metacognitive abilities linked to cognitive mapping would be also innovative, as it is a non-verbal field.

For most, gender equity seems to be an invisible problem, despite efforts to fight side effects from measures taken to combat Covid-19 outbreak. One should bear in mind that cognitive health is greatly influenced by being active, including a good adoption of technology to stay connected in current society. Gender gap in digital technology adoption would be a challenge for our society, where culture might play a crucial role, and cohort effects are expected. In sum, metacognitive skills training could be a starting point to improve the adoption of such technology, to reduce the gap, and ultimately improve the cognitive health of older adults.

## Author Contributions

All authors listed have made a substantial, direct and intellectual contribution to the work, and approved it for publication.

## Conflict of Interest

The authors declare that the research was conducted in the absence of any commercial or financial relationships that could be construed as a potential conflict of interest.
